# Ectoparasitic infestations of the European hedgehog (*Erinaceus europaeus*) in Urmia city, Iran: First report

**Published:** 2013

**Authors:** Tahmineh Gorgani-Firouzjaee, Behzad Pour-Reza, Soraya Naem, Mousa Tavassoli

**Affiliations:** 1 *Department of Pathobiology, Faculty of Veterinary Medicine, Urmia University, Urmia, Iran; *; 2 *Resident in Veterinary Surgery,Department of Surgery and Radiology, Faculty of Veterinary Medicine, Tehran University, Tehran, Iran.*

**Keywords:** Ectoparasite, Hedgehog, Iran, Urmia

## Abstract

Hedgehogs are small, nocturnal mammals that become popular in the world and have significant role in transmission of zoonotic agents. Some of the agents are transmitted by ticks and fleas such as rickettsial agents. For these reason, a survey on ectoparasites in European hedgehog (*Erinaceus europaeus)* carried out between April 2006 and December 2007 from different parts of Urmia city, west Azerbaijan, Iran. After being euthanized external surface of body of animals was precisely considered for ectoparasites, and arthropods were collected and stored in 70% ethanol solution. Out of 34 hedgehogs 23 hedgehogs (67.70%) were infested with ticks (*Rhipicephalus turanicus*). Fleas of the species *Archaeopsylla erinacei *were found on 19 hedgehogs of 34 hedgehogs (55.90%). There was no significant differences between sex of ticks (*p* > 0.05) but found in fleas (*p* < 0.05). The prevalence of infestation in sexes and the body condition of hedgehogs (small, medium and large) with ticks and fleas did not show significant differences (*p* > 0.05). Highest occurrence of infestation in both tick and flea was in June. Among three seasons of hedgehog collection significant differences was observed (*p* < 0.05). The result of our survey revealed that infestation rate in hedgehog was high. According to zoonotic importance of this ectoparasite and ability to transmission of some pathogens, more studies are needed to investigate hedgehog parasites in different parts of Iran.

## Introduction

Hedgehogs are small, nocturnal mammals with bodies covered by spine. The European hedgehog (*Erinaceus europaeus*) is considered as the host of wide variety of pathogens such as viruses, bacteria, fungi and parasites.^1^^-^^3^ For example, the investigation on zoonotic cutaneous leishmaniasis in Iran revealed that, the long-eared hedgehog (*Hemiechinus auritis*) can be reservoir host for this pathogene.^4^ Also it has been suggested that hedgehogs may be a host of *Trichinella* in some parts of the world.^5^ Some of the ectoparasites of the European hedgehog such as tick and flea have zoonotic importance, and they are vector of very pathogen rickettsial agents.^6^^-^^9^

Ticks are blood sucking arthropods, which are ectoparasites of domestic and wild animals. Hedgehogs are infested with several species of ticks.^10^ One of the species can infest hedgehogs is *Rhipicephalus turanicus*. It is wide-spread from Africa to Asia especially around the Caspian sea. This species infests a wide variety of the animals including ruminants, horses, wild carnivores, birds, and small exotic pets like hedgehogs. *Rhipicephalus turanicus *is a three-host tick. The adults generally are common during the late rainy to early dry seasons. The morphologic characters are mild brown color, hexagonal shape of basis capituli, short palp and hipostome. They have pulvilli in the end of legs. This species is very similar to *R. sanguineus* with differences in caudal appendage and genital aperture. In fed males a distinctive broad and protruding caudal appendage is observed.^11^

Hedgehogs are commonly infested with hedgehog flea *Archaeopsylla erinacei*. Also, other species such as cat and dog’s flea are observed. *A. erinacei* has oval head, genal ctenidia (2-3 short spines) and pronotal ctenidia (5-6 spines).^10^ They live mostly in front legs, head, neck and ventral part of body. High infestation results in pruritus, weakening and anemia.^10^ Also, it is mentioned that this species can be vector of rickettsial agents.^6^ However, there is a few information about ectoparasite of hedgehog in different parts of Iran, therefore, the present study aimed to record the ectoparasites species of European hedgehog in Urmia city, Iran.

## Materials and Methods

**Animals.** A total number of 34 hedgehogs (17 males and 17 females) were collected between April 2006 and December 2007 from different regions of Urmia city (45° 37΄ E, 37° 31΄ N), west Azerbaijan, Iran. The animals were euthanized by high dose (6 mg kg^-1^) injection of ketamine (Bioniche Pharma, Lake Forest, IL, USA).^12^ Different parts of body for another objective were examined at necropsy and external surface of body was precisely considered for ectoparasites and collected arthropods were transferred to 70% ethanol solution. Fleas were cleared in 10% potash solution and mounted using routine technique. Identification of the species was confirmed on the basis on light microscopic examination with reference to keys.^10^^,^^11^

**Statistical analysis. **The following parasitological parameters were evaluated: percentage prevalence, the mean intensity and the mean abundance.^13^ Fisher exact test and Whitney U test by SPSS (Version 16.0 for Windows, SPSS Inc., Chicago, IL, USA) were used to compare the prevalence of ectoparasite infestation between the hedgehogs’ body condition, sexes and seasons.

## Results

**Fig. 1:**
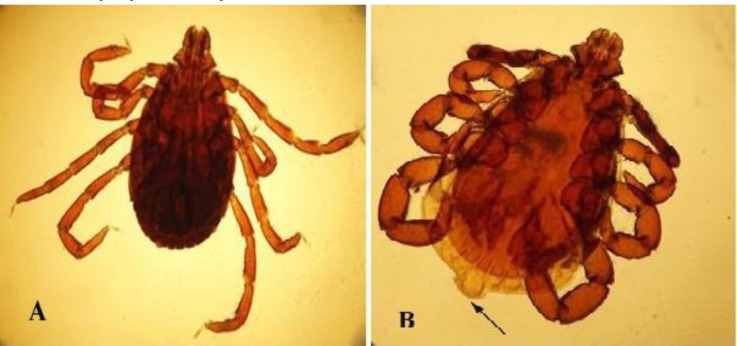
**A)** Female of *R. turanicus* tick. **B)** Male *R. turanicus*, broad caudal appendage (Arrow), (25×).

**Fig. 2:**
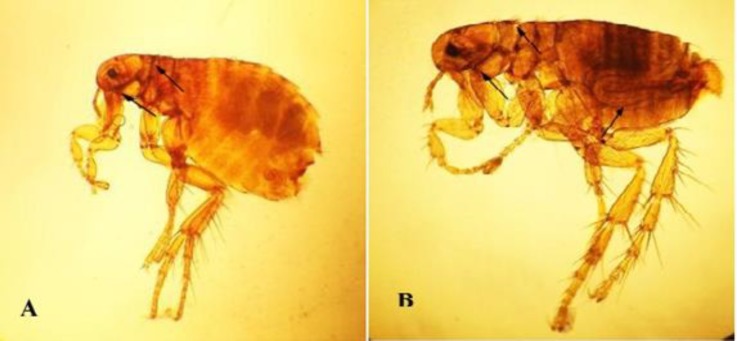
**A** **)** Female hedgehog flea; *A. erinacei*, genal and pronotal ctenidia (Arrow). **B)** Male *A. erinacei*, genal and pronotal ctenidia and aedeagus organ (Arrow), (40×).

A total number of 241 ticks (127 males and 114 females) were collected from hedgehogs. There were no differences in male and female ticks (*p* > 0.05). Out of hedgehogs, 23 were infested with ticks (67.70%). All ticks were belonged to *R. turanicus *species (Fig. 1)*.* Percentage of fed ticks on the body of hedgehogs was 72.20% (67 females and 107 males). Fleas of the species *A. erinacei *were found on 19 hedgehog of 34 (55.90%) examined hedgehog (Fig. 2). The number of infested fleas was 148 (42 males and 106 females), (Table1).

**Table 1.** Prevalence and intensity of ectoparasite species on hedgehogs (n = 34). Data for intensity and abundance are presented as mean ± SD.

**Parasite species**	**Prevalence (%)**	**Intensity**	**Abundance**	**Range**	**Sex ratio**	**Fed ticks (%)**
***Rhipicephalus turanicus***	23 (67.70%)	10.47 ± 7.00	4.35 ± 6.60	1-25	1/1.15	72.20
**Archeopsylla erinacei**	19 (55.90%)	7.78 ± 7.20	7.08 ± 7.60	2-34	1/2.52	-

**Table 2:** Prevalence of ectoparasite infestation in relation to body condition and sex of the hedgehogs (n = 34).

**Parasite species**	**Prevalence (%)**						
	**Body weight**			**Significance**	**Sex**		**Significance**
	Small (n=2)	Medium (n=25)	Large (n=7)		Female (n=17)	Male (n=17)	
***Rhipicephalus turanicus***	-	4 (57.15%)	19 (76.00%)	*p* > 0.05	11 (64.70%)	12 (70.60%)	*p* > 0.05
**Archeopsylla erinacei**	-	17 (68.00%)	2 (28.60%)	*p* > 0.05	10 (58.80%)	9 (52.90%)	*p* > 0.05

Rates of infestation with *R. turanicus* and *A. erinacei* was not different (*p* > 0.05) between small, medium and large hedgehogs. There was no difference (*p* > 0.05) between the sexes of hedgehogs for the rates of infestation with ticks and fleas (Table 2). No difference was seen between female and male ticks (*p* > 0.05), although female and male flea had significant differences (*p* < 0.05).

**Fig. 3:**
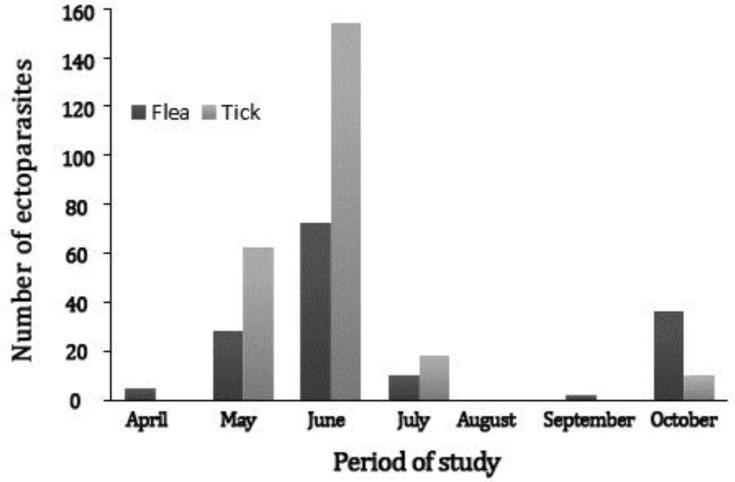
Infection rate of hedgehog fleas and ticks collected from Urmia, Iran.

Highest occurrence of infestation in both tick and flea was in June (Fig. 3). Prevalence of ectoparasite in spring, summer and fall were 90.50%, 50.00%, 14.30% for ticks and 76.20%, 33.30%, 14.30% for fleas, respectively. The significant variations were observed among seasons in both ectoparasite species (*p* < 0.05).

## Discussion

Our results suggest that infestation rate with ticks and fleas were high in hedgehog population. All examined hedgehog were belonged to *E. europaeus *species. Tick species, which was found in the present survey, was *R. turanicus*. In a study which was carried out in north of Iran, *R. turanicus* was reported from hedgehogs (*E. concolor*) with 75.00% infestation rate.^14^ It was mentioned that commonly two tick species are found on the European hedgehogs, the hedgehog tick *Ixodes hexagonus *and the sheep tick *I. ricinus*. However, other species can infest hedgehog.^10^ According to Rahbari *et al.,* *R. turanicus *has commonly presented among ruminants in most parts specially in west of Iran.^14^ The most prevalence of this tick population had been in spring and started from April to May.^15^ Also, in our study, highest prevalence was in June, which had significant differences among seasons, as well. The results of this study was similar to study by Pfaffle *et al*. on tick populations in hedgehogs (*E. europaeus*).^16^ Parallel to our survey in other surveys, no significant differences had been seen between sex of hedgehogs.^16^^,^^23^ Another study in rural region of Tabriz showed that three 14 were infested with *R. appendiculatus.*^17^ Also, other studies were indicated that tick infestation is common in hedgehogs and they are suitable host for several pathogens. In a study, which was carried out in France, European hedgehogs were infested with *R. sanguineus* and 91.70% of ticks were positive for *R. masssiliae*, which is the cause of spotted fever disease. This survey and another investigations suggest that *Rhipicephalus* species are potential vectors and reservoirs for these pathogens.^6^^,^^18^^-^^19^ In addition, another study on African hedgehog (*Atelerix algirus*) and desert hedgehog (*Paraechinus aethiopicus*) in Algeria indicated that examined ticks; *R. sanguineus* (17.50%) and *Haemaphysalis erinacei* (77.00%) were positive for *Rickettsia masssiliae.*^7^ Therefore, these results suggest that horizontal transmission of *Rickettsia* between hedgehog and tick lead to hedgehog to become reservoir host and in the regions which there are close communication between human and these animals, human can be infected. Furthermore, in previous surveys mentioned that *R. turanicus *can act as a vector for *Rickettsia*, *Anaplasma*, *Theileria*, *Babesia,* and arboviruses.^20^^,^^21^ However, hedgehogs are usually infested with *I. hexagonus* and *I. ricinus*, but these ticks were absent in our study.

In the current study, another arthropod, which was found from examined hedgehogs, was *A. erinacei* with infestation rate of 55.80%. The highest infestation rate was in May and June. Commonly, hedgehogs are infested with hedgehog flea; *A. erinacei*. There was no report from hedgehog flea in Iran, before. In the study carried out on the Northern white-breasted hedgehog (*E. roumanicus*) in urban park of central Budapest, Hungary, 99.40% were infected with *A. erinacei* flea.^22^ In an epidemiological survey, which was done on European hedgehogs in Britain, two ectoparasites species *A. erinacei* (8.00%) and *I. hexagonus *(59.00%) were reported.^23^ According to Thamm *et al*., *I.* *hexagonus *and suggested there were association between ectoparasite infestation and urban environment.^24^ In a survey performed on flea infestation in pets and hedgehog revealed that 14.60% of dogs, 5.10% cats and 84.20% hedgehogs were infested with *A. erinacei.*^25^

In a preliminary study on zoonosis and parasitic infection in hedgehogs (*E. europaeus*) in England at the period of ten years infection with fleas (*A. erinacei), *ticks (*I. hexagonus)*, salmonellosis, pseudotuberculosis and helminthes is reported.^3^ According to some investigations on fleas, the most fleas (*A. erinacei*), collected from hedgehogs were positive for *R. felis*. which is the obligatory intra-cellular bacterium, causing a murine typhus like disease in humans.^6^^,^^7^^,^^26^ Human infestation with flea also, has been already reported.^27^

In conclusion, the results of the present study revealed that the infestation rate of ectoparasites in hedgehog population was high and due to zoonotic importance of the transmission of some important pathogens, more studies are needed to find out hedgehog ectoparasites in other parts of Iran.
